# ACTIGRAPHIC MEASURES OF SLEEP AND METABOLIC SYNDROME AMONG OLDER ADULTS

**DOI:** 10.1093/geroni/igad104.1616

**Published:** 2023-12-21

**Authors:** Diefei Chen, Yiwei Yue, Sharmin Hossain, Jill Rabinowitz, Amal Wanigatunga, Luigi Ferrucci, Eleanor Simonsick, Adam Spira

**Affiliations:** Johns Hopkins Bloomberg School of Public Health, Baltimore, Maryland, United States; Johns Hopkins Bloomberg School of Public Health, Baltimore, Maryland, United States; Maryland Department of Health, Linthicum Heights, Maryland, United States; Johns Hopkins Bloomberg School of Public Health, Baltimore, Maryland, United States; Johns Hopkins Bloomberg School of Public Health, Baltimore, Maryland, United States; NNational Institute on Aging, National Institutes of Health, Baltimore; NNational Institute on Aging, National Institutes of Health, Baltimore; Johns Hopkins Bloomberg School of Public Health, Baltimore, Maryland, United States

## Abstract

Self-reported sleep disturbances are associated with obesity and diabetes, but little is known about links between objectively measured sleep and metabolic syndrome (MetS) in later life. We investigated associations between actigraphic sleep parameters and MetS in 406 participants (53.5% women, 20.9% Black) aged 72.8±10.1 years at baseline (range: 50-96) in the Baltimore Longitudinal Study of Aging. Participants completed wrist actigraphy (6.6±1.0 nights) and were classified as having MetS if they had ≥3 of the following: waist circumference ≥102 cm for men, ≥89 cm for women; high-density lipoproteins < 40 mg/dL in men, < 50 mg/dL in women; triglycerides >150 mg/dL; high blood pressure ≥130/85 mmHg; fasting blood glucose ≥100 mg/dL. Overall, 37 participants had MetS at baseline and 27 participants developed MetS over 2.3±2.2 years of follow-up. Cross-sectionally, compared to participants with total sleep time (TST) < 6 hours, those with 6-8 hours TST had lower odds of MetS (OR=0.28, 95%CI 0.13,0.61); higher sleep efficiency (SE) was associated with lower odds of MetS (OR=0.65, 95%CI 0.48,0.89) and longer wake bout length (WBL) was associated with higher odds of MetS (OR=1.47, 95%CI 1.07,2.01). Excluding 37 participants with MetS at baseline, TST >8 hours was associated with faster increases in the likelihood of developing MetS (OR=1.62, 95% CI 1.03, 2.55). Findings link intermediate TST and higher SE to lower odds of MetS, and longer WBL to higher odds of MetS in older adults cross-sectionally; TST >8 hours was associated with higher odds of incident MetS. Further studies are needed to understand mediators of sleep-MetS associations.

